# Transcultural Adaptation and Psychometric Properties of the Persian Version of the Clinical Learning Environment, Supervision, and Nurse Teacher Scale Among Undergraduate Nursing Students

**DOI:** 10.1155/jonm/8822418

**Published:** 2025-08-07

**Authors:** Zeinab Hosseini, Hamid Sharif-Nia, Pardis Rahmatpour, Omolhoda Kaveh

**Affiliations:** ^1^School of Nursing and Midwifery, Mazandaran University of Medical Sciences, Sari, Iran; ^2^Psychosomatic Research Center, Mazandaran University of Medical Sciences, Sari, Iran; ^3^Department of Nursing, Amol School of Nursing and Midwifery, Mazandaran University of Medical Sciences, Sari, Iran; ^4^Health Sciences Research Unit: Nursing (UICISA: E), Nursing School of Coimbra (ESEnfC), Coimbra, Portugal

**Keywords:** clinical learning environment, clinical supervision, nurse teacher, nursing student, psychometric, reliability, validity

## Abstract

**Objectives:** Clinical learning is the core of nursing education. The clinical learning environment, supervision, and nurse teacher (CLES + T) play a significant role in the formation of optimal clinical learning. So, this study aimed to investigate the psychometric properties of the Persian version of the CLES + T scale among undergraduate nursing students.

**Methods:** In this cross-sectional study, 400 nursing students participated from March to December 2023 via a convenience sampling method. The exploratory (*n* = 200), confirmatory factor analysis (*n* = 200), and reliability of the Persian version of CLES + T were assessed. The analyses were conducted using SPSS V.22, AMOS V.27, and JASP V. 18.

**Results:** The exploratory factor analysis extracted 6 factors (28 items), and the scale's overall explained variance was 41.61%. The confirmatory factor analysis indices confirmed the model fit (CFI = 0.90, PNFI = 0.72, IFI = 0.90, TLI = 0.90, and RMSEA = 0.055). The scale had acceptable internal consistency (*α*: 0.64–0.83).

**Conclusion:** The results of this study support the validity and reliability of the Persian version of the CLES + T with 34 items and 5 factors. The role of the nurse teacher and the supervisory relationship were highlighted as key factors in creating a positive clinical learning environment among Iranian nursing students.

## 1. Introduction

The educational environment plays a very important role in the learning of nursing students [1]. The clinical environment offers nursing students a valuable opportunity to apply and refine their nursing skills, as well as to assess the practical effectiveness of their clinical education [2]. The clinical learning environment holds significant importance in nursing clinical education. Within these settings, various forms of communication and interaction, including clinical supervision, management practices, and patient interactions, significantly impact the learning experiences of nursing students [3, 4]. The educational atmosphere within the learning environment significantly influences nursing students' enthusiasm for the profession. Students must have a clinical learning setting that is supportive, respectful, and encouraging, as this is vital for mastering clinical practice skills [5]. The main goals of nursing students' clinical education are achieved through training in the clinical environment, including developing critical thinking and communication skills, learning therapeutic nursing care, effectively participating in care teams, applying ethical principles, and integrating these principles into decision-making daily in the clinical setting [6].

Half a century ago, Iran's nursing system underwent a transformation with the introduction of academic nursing education. However, this shift still faces challenges, including the gap between theory and practice, issues in student recruitment systems, and a lack of coordination between curricula and community health problems [7]. In theory classes, students' education is well structured and follows a planned approach. However, clinical education has always been unpredictable, making it challenging to manage and plan [4, 8]. In Iran, nursing students partake in clinical training overseen by faculty members, but in cases of staffing shortages, clinical nurses also participate in student instruction. Students begin their clinical immersion in the second semester and undergo various internships throughout their 4-year program [9]. Past research has suggested that newly graduated students may not possess the requisite proficiency, understanding, and competencies to deliver sufficient care and address patient requirements, possibly attributable to inadequate clinical knowledge and training [10, 11]. One of the key responsibilities of a nurse educator is to impart theoretical knowledge to nursing students during clinical practice, facilitating the integration of theory and practice [12]. Based on the available literature, factors such as the discrepancy between theoretical coursework and clinical practice, the absence of well-defined clinical training objectives, the high-stress atmosphere of the hospital setting, the reluctance of seasoned instructors to participate in clinical training settings, the lack of empathy between instructors and students, and the lack of authenticity in evaluation processes can contribute to this issue [13]. Given the significance of enhancing clinical education for nursing students, the efficacy of clinical learning is contingent upon the level of organization within the clinical curriculum. By identifying the impediments to effective learning, it becomes imperative to employ a comprehensive and credible investigative tool. Utilizing a reliable tool will facilitate the acquisition of data on the efficient structuring of clinical practice and the caliber of its supervision [14].

There are numerous assessment tools available for evaluating the clinical educational environment of students, including but not limited to Clinical Learning Environment (CLE), Clinical Learning Environment Inventory (CLEI), Clinical Learning Environment and Diagnostic Inventory (CLEDI), Student Evaluation of Clinical Education Environment (SECEE), Clinical Learning Environment and Supervision (CLES) scale, and the clinical learning environment, supervision, and nurse teacher (CLES + T) [15–20]. The CLES + T scale is the sole instrument that comprehensively assesses the clinical learning environment, the managerial responsibilities of nursing, and the role of the clinical instructor concurrently [18]. Initially, in 2002, Sarikoski and his associates developed and validated the CLES scale in Finland. The scale consists of 27 items and is designed to investigate the clinical learning environment from the perspective of students [17]. In 2008, the researcher made significant revisions to the aforementioned scale and introduced the CLES + T scale. This updated version incorporated an additional subscale focusing on the clinical instructor, recognizing their crucial role in the training of clinical students and their impact on the quality of collaboration with nursing instructors. The CLES + T scale comprises 34 items distributed across 5 domains [5]. The scale's validity and reliability have been rigorously established [17, 18]. The psychometric properties of the CLES + T scale have been rigorously assessed across diverse cultural contexts, including Spain, Germany, Greece, Italy, Norway, Sweden, New Zealand, Hong Kong, and China [1, 14, 21–25]. Given the lack of comprehensive and reliable tools for obtaining information about the effective organization of clinical learning environment due to the importance of the issue of clinical education of nursing students on the one hand and the differences in the culture and context governing clinical learning environments in Iran on the other hand, validation and localization of the CLES + T tool seems necessary. The main aim of this study was to investigate the psychometric properties of the Persian version of the CLES + T scale among undergraduate nursing students. The results of this research effort will provide clinical educators with valuable tools to identify learners' needs, enhance their clinical learning experiences, and formulate effective strategies to enrich the overall clinical learning environment.

## 2. Method

### 2.1. Study Design

This cross-sectional study was conducted from March to December 2023 to translate and evaluate the psychometric validity of the Persian version of the CLES + T scale among nursing students of three nursing schools of Mazandaran University of Medical Sciences in northern Iran.

### 2.2. Participants

According to Kellar and Kelvin [26], the minimum sample size for conducting a factor analysis is equal to between 5 and 10 participants per item of the intended instrument. Although the original scale had 34 items, after accounting for attrition, 450 scales were distributed to eligible students, of which 400 were fully completed (response rate = 88%). Second-year undergraduate nursing students and above who were willing to participate in the study and were studying in nursing schools of Mazandaran University of Medical Sciences were included in the study using convenience sampling. After obtaining permission from the faculty officials, the researchers distributed the scales to the students and returned the next day for collection.

### 2.3. Measurement

Data were collected by a paper questionnaire that consisted of two parts: demographic information and the Persian version of CLES + T.

1. The demographic information of students: age, gender, marital status, and the academic year.
2. The Persian version of CLES + T: the original version of CLES + T was developed in Finland in 2008 by Saarikoski and Leino-Kilpi [17]. Initially, the researchers designed the Clinical and Supervisory Learning Environment (CLES) survey tool in 2002 and 2008, they expanded it to include the role of the nursing instructor. The Cronbach's alpha values for this scale ranged from 0.77 to 0.96 for individual domains, with a total Cronbach's alpha of 0.9. The CLES + T is a self-report scale with 34 items organized into 5 domains: supervisory relationship, the role of the nursing instructor, the educational atmosphere in the department, leadership style of the department manager, and nursing delivery in the department. Respondents use a 5-point Likert scale to indicate their level of agreement, ranging from 1 for *complete disagreement* to 5 for *complete agreement* [18].

### 2.4. Translation

After receiving approval from the original developers of the “CLES + T scale,” the translation and cultural adaptation process was conducted according to the Saarikoski et al.'s model [18]. Forward and back translation: according to this model, two Iranian translators proficient in both Persian and English languages and culture independently translated the scale from English to Persian. Subsequently, the Persian translations were consolidated into a singular translation by skilled professionals. Following this, the scale was independently translated back into English by two Persian–English translators. The translation was then confirmed by a panel of experts proficient in both languages, who were unaware of the original content of the scale [27].

### 2.5. Content Validity

To validate the content, the CLES + T scale was administered to 10 nursing faculty members and evaluated the accuracy of item assignment, wording, and representativeness. The experts also assessed the necessity of the items, and the content validity ratio (CVR) and content validity index (CVI) were calculated, with the minimum acceptable value being 0.62 for CVR and 0.7 for CVI [28].

### 2.6. Construct Validity

The construct validity of the CLES + T scale was conducted through both exploratory factor analysis (EFA) and confirmatory factor analysis (CFA). The study dataset was randomly divided into two parts. The first dataset (*n* = 200) was utilized for EFA, employing maximum likelihood (ML) and Promax rotation in SPSS Version 22. The study sample and model's appropriateness were assessed using Kaiser–Meyer–Olkin (KMO) and Bartlett's test of sphericity for factor analysis. The number of factors was determined using Horn's parallel analysis, and factor extraction relied on eigenvalues > 1 and commonalities > 0.2 [29, 30]. The analysis determined the factor structure by calculating eigenvalues, which represent the variance in each item accounted for by the factor. The percentage of total variance explained by each factor was calculated by dividing the eigenvalue by the total number of items [31].

The structural factors were evaluated through a CFA using the ML method and commonly used goodness of fit indices. The CFA evaluation was conducted on the second dataset (*n* = 200) using AMOS software Version 27. Model fitness was assessed based on the root mean square of error of approximation (RMSEA < 0.08), Comparative Fit Index (CFI > 0.90), Parsimonious Normed Fit Index (PNFI > 0.50), Incremental Fit Index and Tucker–Lewis Index (IFI and TLI > 0.90), and CMIN/DF (< 3) [29, 32].

### 2.7. Convergent and Discriminant Validity

To assess convergent validity, the study examined whether the construct composite reliability (CR) was greater than 0.6 and the average variance extracted (AVE) was greater than 0.5 but less than its respective CR [33].

For discriminant validity, the maximum shared squared variance (MSV) for each construct should be less than AVE [34]. Furthermore, the Heterotrait–Monotrait (HTMT) ratio was used to evaluate discriminant validity with the JASP software v.18, and the values had to be less than 0.85 [35].

### 2.8. Reliability

The CLES + T scale's internal consistency was evaluated by ensuring that both Cronbach's alpha (*α*) (between 0.6 and 0.8 is acceptable) [36] and McDonald's omega (Ω) (greater than 0.6 is acceptable) [37]. The CR (which replaces Cronbach's alpha coefficient in structural equation modeling) [38] and maximal reliability H (exceeding 0.7 was considered favorable) were also employed [29].

## 3. Result

The results of the study revealed that out of the 400 nursing students surveyed, the average age was 21.55 years (±1.7). The majority of the respondents were male (52.5%), single (97.25%), and second year (47/75%). The other demographic information of the participants is presented in Table 1.

In assessing content validity based on CVI and CVR, all items achieved acceptable values. The results of EFA indicated that the KMO was 0.897, showing a high level of sampling adequacy for factor analysis. In addition, Bartlett's test of sphericity produced a significant result (*χ*^2^ = 4412.745), further supporting the data's suitability for factor analysis.

In the EFA phase, six factors consisting of 28 items were extracted, namely, “Role of nurse teacher” (7 items), “Supervisory relationship” (5 items), “Leadership style of the ward manager” (5 items), “Pedagogical atmosphere” (5 items), “Training meeting” (3 items), and “Premises of nursing on the ward” (3 items).

**Figure 1:**
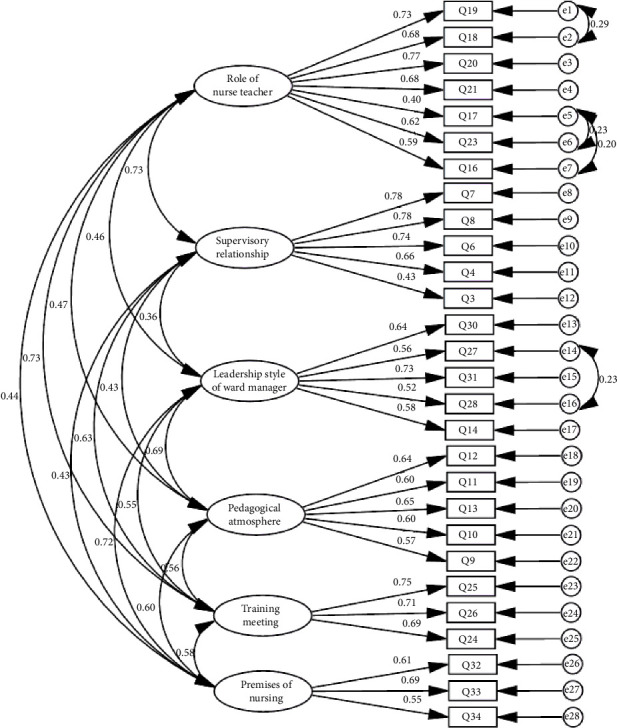
The CFA model of the Persian version of the CLES + T scale (*n* = 200).

Subsequently, a ML CFA involving 200 participants was conducted to validate the factor structure derived from EFA (see Table 2). The results of the CFA revealed that the 6-factor model fit statistics were as follows: *χ*^2^ (331) = 727.91, *p* < 0.001, *χ*^2^/df = 2.199, CFI = 0.90, PNFI = 0.72, IFI = 0.90, TLI = 0.90, and RMSEA = 0.055 (90% CI: 0.049–0.060) (see Figure 1).

According to the convergent validity assessment indices, the AVE of the five of six factors (except factor 5: training meeting) was lower than 0.5; however, the CR of all factors was higher than 0.6 and higher than the AVE. The HTMT matrix confirmed the divergent validity of six factors of scale with results below 0.85 (Table 3).

The acceptable internal consistency was observed for all factors, with Cronbach's alpha and omega coefficients greater than 0.6. The CR and maximum reliability were excellent for the five out of six factors in the model. However, the sixth factor did not meet the acceptable threshold (Table 3).

## 4. Discussion

This study, which focused on translating and validating the CLES + T scale, performed EFA and CFA on a sample of nursing students. Based on the results of factor analysis, six factors were extracted, and CFA was used and confirm the goodness of fit of the Persian version of the CLES + T scale.

The total explained variance of the Persian version of the CLES + T scale was 41.61%. The reported variance of this scale in different translations studies from the highest to the lowest were Slovenia: 67.69% [39], Greek: 67.40% [14], Spain: 66.4% [21], Norway: 64% [24], Italy: 62.72% [23], and Sweden: 60.02% [22]. Although it can be said that one of the reasons for this difference is cultural and contextual differences in nursing education and clinical practice in Iran may influence how students perceive and respond to items on the CLES + T scale; however, the key reason for this discrepancy may be the use of different factor extraction methods across studies. While we used ML extraction, other studies used principal component analysis (PCA) or principal axis factoring (PAF), which typically results in higher explained variance because it includes both common and unique variance, whereas ML focuses on estimating only the shared variance among items [40].

In this study, the greatest values of the explained variance belonged to the “*role of nurse teacher*” factor (variance = 10.11%), same as the Slovenian version of the CLES + T scale with 15.18% explained variance [39]. The first extracted factor with seven items was “*the role of nurse teachers*” related to nursing teachers' capability to meet learning goals in the clinical environment. One of the main roles of nursing professors is clinical teaching. The quality teaching of a professor improves the cognitive and procedural skills of nursing students [41].

The second factor was supervisory relationship (variance = 9.10%), which was the first factor in Italian [23], Slovenian [39], and Swedish versions [22] of the CLES + T scale. The six items of this subscale were related to appropriate supervision and respectful relationships in clinical settings. A good relationship based on mutual respect, ethics of responsibility, and professional behavior between nursing students and supervisors can lead to creating a sense of belonging to the nursing profession and improve nursing students' learning and professional development [42].

The third factor was labeled as leadership style of the ward manager (variance = 6.3%) with five items reflecting a ward manager who appreciates the efforts of nurses and provides good feedback to nursing students. Manager with effective leadership increases nurses' satisfaction, improves nursing care, and could be a good model for nursing students [43].

The pedagogical atmosphere was extracted as a fourth factor of the CLES + T scale in this study. This subscale has five items (variance = 6%) that addressed the positive educational atmosphere that creates learning opportunities for nursing students. Acceptance of nursing students by nurses and their participation in discussions increases students' motivation [44]. The pedagogical atmosphere was the first and important factor in Italian version of the CLES + T scale [23].

The fifth subscale was a training meeting with three items (variance = 5.10%). In the original scale and different translations, these three items belonged to the “role of nurse teacher” subscale; however, based on the nursing students' responses in this study, these three items create a new subscale that is especially related to the meeting that students, nurses, and nurse teacher had in the clinical environment.

Finally, the last subscale was premises of nursing on the ward with three items (variance = 5%). There was a challenge related to this subscale. Although each item loaded acceptably in EFA and CFA, the interitem correlations were not strong enough to yield high reliability, suggesting the items may not cohere tightly as a single construct in this context. These differences likely reflect contextual and cultural variations in nursing education and clinical environments in Iran.

According to the results of internal consistency indices such as Cronbach's alpha and McDonald's omega, the six factors of the Persian version of the CLES + T scale demonstrated acceptable internal consistency. Although the subscale “Premises of Nursing on the Ward” showed lower-than-expected reliability (CR = 0.65 and MaxR(H) = 0.66), we decided to retain it in the model. One reason could be due to fewer items of this factor, and as we discussed before, the construct itself might not be a recognized or meaningful concept in the Iranian nursing education system, reducing coherence among the items. Future research should consider revising or expanding the items to enhance reliability.

The divergent validity of the scale was confirmed based on the HTMT matrix. Although the AVE for the five factors was less than 0.5, Fornell and Larcker [33] recommended that if the AVE is less than 0.5, CR is greater than 0.6 for a psychological construct, and the convergent validity of the construct can be established. Indeed, AVE is a strict measure of convergent validity and a more conservative measure than CR. Hence, based on CR, convergent validity for constructs was achieved.

### 4.1. Limitation

This study had some limitations. The cross-sectional design of the study does not allow for the observation of changes over time or the establishment of causal relationships between variables. The use of a self-report questionnaire introduces the potential for response bias. The reliance on ML estimation assumes multivariate normality, which may not fully hold for all items. In addition, another potential source of bias is the variability in clinical learning environments across different hospitals, and factors such as the hospital size, type of ward, supervision quality, and differences in clinical practices may influence students' perceptions, potentially affecting the comparability of responses. Participants might have provided socially desirable answers or may not accurately recall their experiences, which could influence the validity of the results. The study was conducted with a sample of 400 nursing students from a specific region in Iran, which may limit the generalizability of the findings to other cultural or educational settings.

## 5. Conclusion

The validity and reliability of the Persian version of the CLES + T scale, with 28 items and 6 domains, were verified. The nurse teacher's role and the supervisory relationship were pointed out as the main factors in the fulfillment of a positive clinical learning environment. The validated Persian version of the CLES + T scale provides educators and nursing administrators with a reliable to evaluate and monitor students' perceptions of the clinical learning environment. Results from this scale can help identify strengths and weaknesses in supervision quality, pedagogical atmosphere, and the role of nurse educators. These insights can inform targeted interventions, such as structured mentorship programs, workshops for clinical instructors, and reforms in clinical placement policies, all aimed at enhancing the quality and consistency of clinical education in Iran. The study also gives scope for other researchers to look into the use of the CLES + T scale in other cultures and other study designs, such as a longitudinal study. From an educational perspective, the findings underscore the importance of fostering supportive and pedagogically rich clinical environments. Nursing faculties can use the CLES + T scale data to tailor training programs for clinical instructors and nurse teachers, promote student-centered learning approaches, and create feedback mechanisms for continuous improvement. Incorporating regular assessment of clinical environments into nursing curricula could help align educational strategies with students' needs, ultimately enhancing clinical learning outcomes and professional readiness.

## Figures and Tables

**Table 1:** Demographic characteristics of the participants (*n* = 400).

Variables	*N* (%)	
Gender	Male	210 (52.5%)
	Female	190 (47.5%)

Marital status	Single	389 (97.25%)
	Married	11 (2.75%)

Academic year	2	191 (47/75%)
	3	117 (29.25%)
	4	92 (23%)

**Table 2:** The results of exploratory factor analysis of the Persian version of the CLES + T scale (*n* = 200).

Items	Factor loading	*h* ^2∗^
*Role of nurse teacher (ƛ = 2.63, variance = 10.11%)*		
Q19. The NT was capable of operationalizing the learning goals of this placement	0.847	0.309
Q18. In my opinion, the NT was capable of integrating theoretical knowledge and the everyday practice of nursing	0.792	0.229
Q20. The NT helped me to reduce the theory-practice gap	0.643	0.443
Q21. The NT was like a member of the nursing team	0.550	0.557
Q17. The ward can be regarded as a good learning environment	0.465	0.691
Q23. The NT and the clinical team worked to support my learning	0.428	0.645
Q16. The learning situations were multidimensional in terms of content	0.411	0.329

*Supervisory relationship (ƛ = 2.36, variance = 9.10%)*		
Q7. Mutual respect and approval prevailed in the supervisory relationship	0.888	0.371
Q8. The supervisory relationship was characterized by a sense of trust	0.809	0.403
Q6. There was a mutual interaction in the supervisory relationship	0.595	0.485
Q4. Overall, I am satisfied with the supervision I received	0.515	0.414
Q3. I continuously received feedback from my supervisor	0.434	0.343
Q1. My supervisor showed a positive attitude toward supervision	0.332	0.366

*Leadership style of the ward manager (ƛ = 1.63, variance = 6.30%)*		
Q30. The effort of individual employees was appreciated	0.694	0.289
Q27. The ward manager (WM) regarded the staff on her/his ward as a key resource	0.632	0.621
Q31. The ward's nursing philosophy was clearly defined	0.510	0.670
Q28. The WM was a team member	0.505	0.575
Q14. The staff learned to know the students by their names	0.489	0.471

*Pedagogical atmosphere (ƛ = 1.53, variance = 6.00%)*		
Q12. There was a positive atmosphere on the ward	0.655	0.407
Q11. During staff meetings (e.g., before shifts), I felt comfortable taking part in the discussions	0.597	0.494
Q13. The staff were generally interested in student supervision	0.543	0.673
Q10. I felt comfortable going to the ward at the start of my shift	0.540	0.491
Q9. The staff were easy to approach	0.404	0.424

*Training meeting (ƛ = 1.31, variance = 5.10%)*		
Q25. In our common meetings, I felt that we were colleagues	0.815	0.340
Q26. The focus of the meetings was on my learning needs	0.583	0.449
Q24. The common meetings between me, my mentor, and NT were a comfortable experience	0.555	0.488

*Premises of nursing on the ward (ƛ = 1.30, variance = 5%)*		
Q32. Patients received individual nursing care	0.732	0.568
Q33. There were no problems in the information flow related to patient care	0.519	0.390
Q34. Documentation of nursing (e.g., nursing plans and daily recording of nursing procedures) was clear	0.487	0.297

*Note:λ*, eigenvalue.

^∗^ *h* ^2^, item communality.

**Table 3:** The results of the convergent, discriminant validity, and reliability of the Persian version of the CLES + T scale.

Indices factors	Convergent and discriminant validity				Internal consistency		HTMT analysis					
	CR	AVE	MSV	MaxR	*α*	Ω	1	2	3	4	5	6
1	0.851	0.454	0.601	0.865	0.836	0.833	1					
2	0.835	0.465	0.601	0.860	0.821	0.806	0.756	1				
3	0.745	0.372	0.539	0.758	0.748	0.747	0.513	0.375	1			
4	0.752	0.378	0.491	0.754	0.748	0.749	0.517	0.511	0.694	1		
5	0.769	0.526	0.553	0.770	0.756	0.762	0.734	0.659	0.562	0.582	1	
6	0.655	0.389	0.539	0.662	0.645	0.652	0.489	0.477	0.702	0.606	0.601	1

*Note:* Factors' label: (1) role of nurse teacher; (2) supervisory relationship; (3) leadership style of the ward manager; (4) pedagogical atmosphere; (5) training meeting; and (6) premises of nursing on the ward.

## Data Availability

The data that support the findings of this study are available from the corresponding author upon reasonable request.
